# Refractory Obstetric Hemorrhage As the Initial Presentation of Acquired Hemophilia: A Case Report on the Diagnostic Limitations of Viscoelastic Testing

**DOI:** 10.7759/cureus.117012

**Published:** 2026-09-27

**Authors:** Maria Alaciel Galvan Merlos, Maria Dioselina Ruiz Barrera, Cristina Elizabeth Madera Maldonado, Liliana Ponce Baños, Jorge Omar Castro Meza, Lourdes Bautista Martinez, Leticia Guerrero Navarrete

**Affiliations:** 1 Critical Care Medicine, Hospital Regional de Alta Especialidad de Ixtapaluca, Ixtapaluca, MEX; 2 Internal Medicine, Hospital Regional de Alta Especialidad de Ixtapaluca, Ixtapaluca, MEX; 3 Intensive Care Medicine, Hospital Regional de Alta Especialidad de Ixtapaluca, Ixtapaluca, MEX; 4 Hematology, Hospital Regional de Alta Especialidad de Ixtapaluca, Ixtapaluca, MEX

**Keywords:** acquired haemophilia a, factor viii inhibitor assay, intensive & critical care, pospartum, thromboelastography (teg)

## Abstract

Acquired hemophilia A is a rare autoimmune bleeding disorder caused by the development of autoantibodies against coagulation factor VIII. It is frequently associated with the postpartum period and may present as severe and persistent bleeding in patients without a prior history of coagulopathy, often leading to diagnostic delay.

We report the case of a 26-year-old woman in the postpartum period following cesarean section for severe preeclampsia, who developed refractory hemorrhage despite multiple surgical interventions, including exploratory laparotomies, hematoma drainage, and abdominal packing. Laboratory evaluation revealed an isolated prolongation of activated partial thromboplastin time. Mixing studies showed incomplete correction, raising suspicion of a circulating inhibitor. Further testing confirmed severely reduced factor VIII activity and the presence of a factor VIII inhibitor. Serial thromboelastography demonstrated profound hypocoagulability with impaired thrombin generation and episodes of hyperfibrinolysis.

The patient was treated with bypassing agents and combined immunosuppressive therapy, resulting in progressive reduction of inhibitor levels and recovery of factor VIII activity, ultimately achieving complete hematologic remission.

This case highlights the diagnostic challenges of acquired hemophilia A in the postpartum setting and emphasizes the importance of early recognition of isolated coagulation abnormalities. Prompt diagnosis and a coordinated multidisciplinary approach are essential for effective bleeding control and inhibitor eradication in critically ill patients.

## Introduction

Obstetric hemorrhage remains one of the leading causes of maternal mortality worldwide, accounting for approximately 25% of direct maternal deaths, with an even greater impact in middle-income countries where diagnostic and therapeutic limitations may worsen clinical outcomes [1]. Although most cases are attributed to structural causes - primarily uterine atony, obstetric trauma, or retained placental tissue - a less frequent but clinically devastating subset of cases arises from primary hemostatic disorders that are not promptly recognized [2-4].

Among these rare conditions, acquired hemophilia A (AHA) represents a particularly important entity. It is an autoimmune bleeding disorder caused by neutralizing autoantibodies directed against coagulation factor VIII, which critically interfere with the formation of the intrinsic tenase complex and impair the amplified generation of thrombin [5,6]. Unlike congenital hemophilia, AHA typically affects previously healthy adults without a prior bleeding history, with an estimated incidence of 1-1.5 cases per million per year [6,7]. In young women, approximately 7% to 21% of cases occur during pregnancy or the postpartum period, most commonly within the first four months after delivery [5,7].

Postpartum acquired hemophilia is of particular relevance in obstetric critical care, as it often manifests as bleeding that is disproportionate to the surgical procedure performed, including large abdominal wall hematomas, deep muscular bleeding, or persistent surgical wound hemorrhage despite adequate surgical technique and appropriate transfusion support [8]. Unlike typical obstetric hemorrhage, it is usually not associated with significant thrombocytopenia or hypofibrinogenemia, which can delay diagnostic suspicion in clinical scenarios where initial evaluation is focused primarily on mechanical causes of bleeding [3,9].

In this context, it is crucial to recognize that, in the presence of inhibitors, the interpretation of coagulation tests has important limitations. Both conventional coagulation assays and viscoelastic tests (thromboelastography (TEG) and rotational thromboelastometry (ROTEM)) may remain abnormal as long as the circulating inhibitor persists, even after administration of hemostatic therapies such as recombinant activated factor VII (rFVIIa). This is explained by the fact that bypassing agents restore thrombin generation independently of the intrinsic tenase complex without modifying the underlying immune-mediated mechanism. Therefore, laboratory improvement correlates more closely with inhibitor eradication than with the immediate effect of hemostatic interventions. Furthermore, dilution and correction studies play a critical diagnostic role, as FVIII inhibitors exhibit complex, concentration-dependent kinetics. Unlike simple factor deficiencies, where dilution leads to proportional prolongation of clotting times, in the presence of an inhibitor the activated partial thromboplastin time (aPTT) may paradoxically shorten as the inhibitor concentration decreases, resulting in characteristic non-linear patterns. Additionally, these inhibitors may demonstrate time- and temperature-dependent behavior, and their proper detection requires incubation at 37°C, which has direct implications for the interpretation of mixing studies [10].

The classical laboratory finding in AHA is isolated prolongation of aPTT with a normal prothrombin time (PT). However, in the context of massive obstetric hemorrhage, interpretation may be confounded by hemodilution, repeated transfusions, and dynamic changes in fibrinogen levels [2,11].

Viscoelastic testing methods such as TEG and ROTEM have become central tools in the management of obstetric hemorrhage, allowing goal-directed transfusion strategies and reducing unnecessary blood product administration [6,9,12]. Nevertheless, their ability to detect isolated intrinsic pathway deficiencies is limited. Prolongation of reaction time (R) or clotting time (CT) reflects delayed thrombin generation but does not distinguish between FVIII deficiency, hypofibrinogenemia, or dilutional coagulopathy [12].

Clinical relevance

Refractory obstetric hemorrhage secondary to acquired hemophilia represents a major diagnostic challenge in obstetric critical care. The isolated interpretation of viscoelastic tests may be insufficient to identify the underlying etiology when a specific FVIII inhibitor is present. Clinical suspicion in the presence of isolated aPTT prolongation and disproportionate bleeding can significantly alter the therapeutic approach and prevent repeated surgical interventions.

The present case illustrates the complex interaction between postpartum immunological changes, altered hemostasis, and the diagnostic limitations of viscoelastic testing, emphasizing the importance of integrating clinical findings, conventional coagulation assays, and specific factor studies in order to achieve an accurate and timely diagnosis.

This paper was published earlier on the Research Square preprint server on April 07, 2026 (DOI: 10.21203/rs.3.rs-9228302/v1).

## Case presentation

A 26-year-old woman, previously healthy and without any personal or family history of known bleeding disorders, primigravida, developed a pregnancy complicated during the third trimester by preeclampsia with severe features. Due to progressive maternal clinical deterioration and the associated obstetric risk, termination of pregnancy was performed by Kerr-type cesarean section at a secondary-level hospital.

During the surgical procedure, uterine atony refractory to conventional uterotonic management was documented. As a result, a B-Lynch uterine compression suture was performed as a conservative strategy for the control of obstetric hemorrhage. Despite this intervention, the patient developed persistent bleeding in the immediate postoperative period, prompting an exploratory laparotomy in order to identify and control the hemorrhagic source. During surgical exploration, an expansive abdominal wall hematoma was identified and surgically drained.

Given the persistence of bleeding and the difficulty in achieving definitive hemostasis, abdominal packing was implemented as a damage control surgery strategy, aiming to obtain temporary hemostasis and allow hemodynamic stabilization. Despite these initial interventions, the patient continued to show clinical deterioration and persistent hemorrhage, which led to her referral to the Hospital Regional de Alta Especialidad de Ixtapaluca for multidisciplinary management in the Adult Intensive Care Unit, due to the complexity of her condition and the need for advanced supportive care.

Admission to the intensive care unit

The patient was admitted to the Adult Intensive Care Unit on ICU Day 1 in the context of complicated postoperative puerperium following cesarean section, with a recent history of multiple abdominal interventions due to persistent bleeding. Before transfer, she had undergone Kerr-type cesarean section, B-Lynch uterine compression suture, exploratory laparotomy, abdominal wall hematoma drainage, and abdominal packing as damage-control surgery.

Upon admission, she was hemodynamically compensated under close monitoring. However, clinical and laboratory findings showed significant anemia secondary to recent obstetric hemorrhage, persistent bloody output through abdominal drains, and abnormal coagulation parameters, particularly isolated prolongation of aPTT.

At this early stage, the coagulation abnormalities were initially interpreted in the context of coagulopathy associated with massive hemorrhage, previous transfusion therapy, and possible coagulation factor consumption, all of which are commonly encountered in severe obstetric bleeding. Nevertheless, the persistence of isolated activated partial thromboplastin time prolongation, together with preserved fibrinogen and relative preservation of prothrombin time, later raised suspicion of an acquired intrinsic pathway coagulation disorder.

Clinically, the patient presented signs of systemic inflammatory response secondary to surgical trauma, along with persistent bloody drainage from previously placed surgical drains. In light of these findings, multidisciplinary management in the critical care setting was initiated, including close hemodynamic monitoring, serial evaluation of hematologic and coagulation parameters, and ongoing surgical assessment due to the possibility of requiring additional interventions to control the bleeding.

Surgical course during hospitalization

During her ICU stay, the patient experienced a complex clinical course characterized by persistent abdominal wall bleeding, progressive anemia, and ongoing hemostatic abnormalities, leading to multiple surgical reinterventions.

On ICU Day 2, an exploratory laparotomy with abdominal unpacking and drain placement was performed. Eight intra-abdominal surgical compresses previously placed at the referring hospital were removed. Systematic exploration revealed no active intra-abdominal bleeding. The uterus showed an intact hysterotomy with a functional B-Lynch compression suture and no evidence of uterine hemorrhage. The adnexa appeared macroscopically normal. Quadrant-by-quadrant exploration did not reveal an active bleeding source. A Penrose drain was placed toward the pelvic cavity, and Blake drains were placed in the subcutaneous tissue. Estimated blood loss was approximately 400 mL.

Despite this intervention, the patient continued to show hematologic deterioration and clinical evidence of ongoing bleeding. On ICU Day 6, a second urgent exploratory laparotomy was performed. An estimated hemoperitoneum of approximately 200 mL was documented, without a clear intraperitoneal bleeding source. However, a large abdominal wall hematoma dissecting through the subcutaneous adipose tissue was identified, associated with active bleeding from parietal planes. Hematoma drainage, local hemostatic control, supra-aponeurotic packing, and drain placement were performed. Estimated blood loss during this intervention was approximately 1000 mL.

On ICU Day 9, a third exploratory laparotomy was performed, including abdominal unpacking, surgical debridement, and drainage of an infected abdominal wall hematoma. Friable parietal tissue and active superficial bleeding were identified. Hemostasis was achieved using direct suturing and topical hemostatic agents, followed by supra-aponeurotic repacking and drain placement.

On ICU Day 11, another exploratory laparotomy with unpacking and surgical lavage was performed. Active bleeding from the right epigastric artery was identified and controlled by surgical ligation. Extensive lavage and antisepsis were performed, followed by drain placement and delayed closure. During a second surgical stage on the same ICU day, hemoperitoneum and a dissecting subcutaneous hematoma were documented. Clot evacuation, targeted hemostasis, abdominal lavage, and bilateral drain placement were carried out. The patient required intraoperative transfusion with packed red blood cells and fresh frozen plasma.

These multiple surgical interventions reflected the difficulty of hemorrhage control in the setting of an underlying inhibitor-mediated hemostatic disorder that had not yet been confirmed at that point, as summarized in Table 1.

**Table 1:** Chronological summary of surgical interventions during the patient’s clinical course. Procedures performed at the referring hospital and subsequent reinterventions during ICU hospitalization are summarized. Initial interventions were directed at obstetric hemorrhage control, whereas later procedures addressed hemorrhagic and infectious complications of the abdominal wall.

Phase/Hospital Day	Intervention	Indication	Objective
Pre-ICU period, referring hospital	Kerr cesarean section, B-Lynch uterine compression suture, exploratory laparotomy, abdominal wall hematoma drainage, and abdominal packing as damage-control surgery	Severe preeclampsia complicated by refractory uterine atony and persistent obstetric hemorrhage with expanding abdominal wall hematoma	Obstetric resolution and initial hemorrhage control
ICU Day 2	Exploratory laparotomy, removal of abdominal packing, and drain placement	Persistent bleeding requiring abdominal re-exploration	Abdominal cavity assessment and hemostatic control
ICU Day 6	Exploratory laparotomy, hematoma drainage, and supra-aponeurotic packing	Acute post-hemorrhagic anemia and suspected ongoing bleeding	Control of abdominal wall bleeding
ICU Day 9	Exploratory laparotomy, removal of packing, surgical debridement, infected hematoma drainage, and repacking	Infected abdominal wall hematoma	Local infection control and hemostasis
ICU Day 11	Exploratory laparotomy, removal of packing, infected hematoma drainage, ligation of the right epigastric artery, and delayed closure	Persistent parietal bleeding and infected hematoma	Definitive control of the bleeding source
ICU Day 11, second surgical stage	Exploratory laparotomy, surgical lavage, hemostasis, and drain placement	Hemoperitoneum and bleeding within subcutaneous tissue planes	Hemostatic control and clot evacuation
ICU Day 38	Non-excisional debridement and surgical wound irrigation	Wound dehiscence with abscesses and devitalized tissue	Local septic control and preparation of the surgical bed
ICU Day 41	Surgical lavage and repeat abdominal wall debridement	Persistent abdominal wall infection	Infection control and open wound management

Infectious complications of the abdominal wall

After partial control of bleeding and progressive hemodynamic stabilization, the patient developed infectious complications of the abdominal wall, likely favored by multiple surgical interventions, previous abdominal packing, surgical drains, and extensive tissue injury.

During follow-up, abdominal wound dehiscence was documented, associated with purulent discharge, necrotic tissue, and multiple subcutaneous abscess collections, consistent with deep surgical site infection.

On ICU Day 38, the patient underwent non-excisional surgical debridement and extensive wound irrigation. Purulent material, devitalized tissue, and multiple parietal abscesses were identified. Surgical management included abundant saline irrigation, antisepsis, removal of devitalized tissue, and open wound management with sterile gauze packing and compressive dressing.

On ICU Day 41, repeat surgical lavage and local wound management were performed because of persistent tissue sloughing and purulent secretion. These interventions allowed progressive control of the infectious process and stabilization of the abdominal wound, in parallel with hematologic and immunologic treatment of the underlying hemostatic disorder.

Hemostatic evaluation using thromboelastography (TEG)

During the patient’s stay in the Intensive Care Unit, serial hemostatic monitoring was performed using thromboelastography (TEG) in order to dynamically evaluate clot formation, stability, and lysis in the context of persistent bleeding and multiple surgical interventions. This tool provided a global assessment of coagulation beyond conventional laboratory tests, which is particularly valuable in critically ill patients with complex hemorrhagic conditions.

Early studies performed during the initial phase of hospitalization revealed severe abnormalities compatible with profound hypocoagulability, primarily characterized by marked prolongation of the reaction time (R), reflecting deficient thrombin generation. In addition, prolongation of the clot formation time (K) and a reduction in the alpha angle (α) were observed, findings that indicated a significant delay in the amplification phase of coagulation and impaired fibrin network formation.

In one of the TEG studies performed during the period of greatest hemostatic instability, an extremely prolonged R time of 1193 minutes was documented, accompanied by a markedly negative coagulation index, suggesting near-complete inability to initiate clot formation. This viscoelastic pattern was inconsistent with the expected findings in purely dilutional or consumptive coagulopathy, raising suspicion of an additional pathological mechanism affecting the coagulation cascade.

Subsequent evaluations also revealed episodes of marked hyperfibrinolysis, with LY30 values exceeding 90%, indicating accelerated clot degradation once formed. This finding was particularly relevant, as it suggested significant instability of the hemostatic system and an additional contribution to the persistence of clinical bleeding.

Longitudinal interpretation of the TEG studies demonstrated a dynamic evolutionary pattern. In the early stages, abnormalities were predominantly related to impaired thrombin generation, whereas later phases showed evidence of clot instability and increased fibrinolysis. After initiation of immunosuppressive therapy and targeted hemostatic treatment, viscoelastic studies demonstrated progressive improvement in coagulation parameters, including gradual reduction of the R time, normalization of the alpha angle, and recovery of maximum amplitude (MA), reflecting restoration of clot formation capacity and mechanical stability.

These viscoelastic findings, together with the persistent isolated prolongation of aPTT in conventional coagulation tests, raised suspicion for an acquired inhibitor-mediated coagulation disorder, which prompted the performance of specific coagulation factor studies.

Overall, the viscoelastic evolution allowed dynamic documentation of the transition from a phase of severe inhibition of thrombin generation toward progressive restoration of hemostatic function, illustrating both the complex pathophysiology of acquired hemophilia and the value of thromboelastography in monitoring therapeutic response in critically ill patients, as shown in Figure 1.

**Figure 1:**
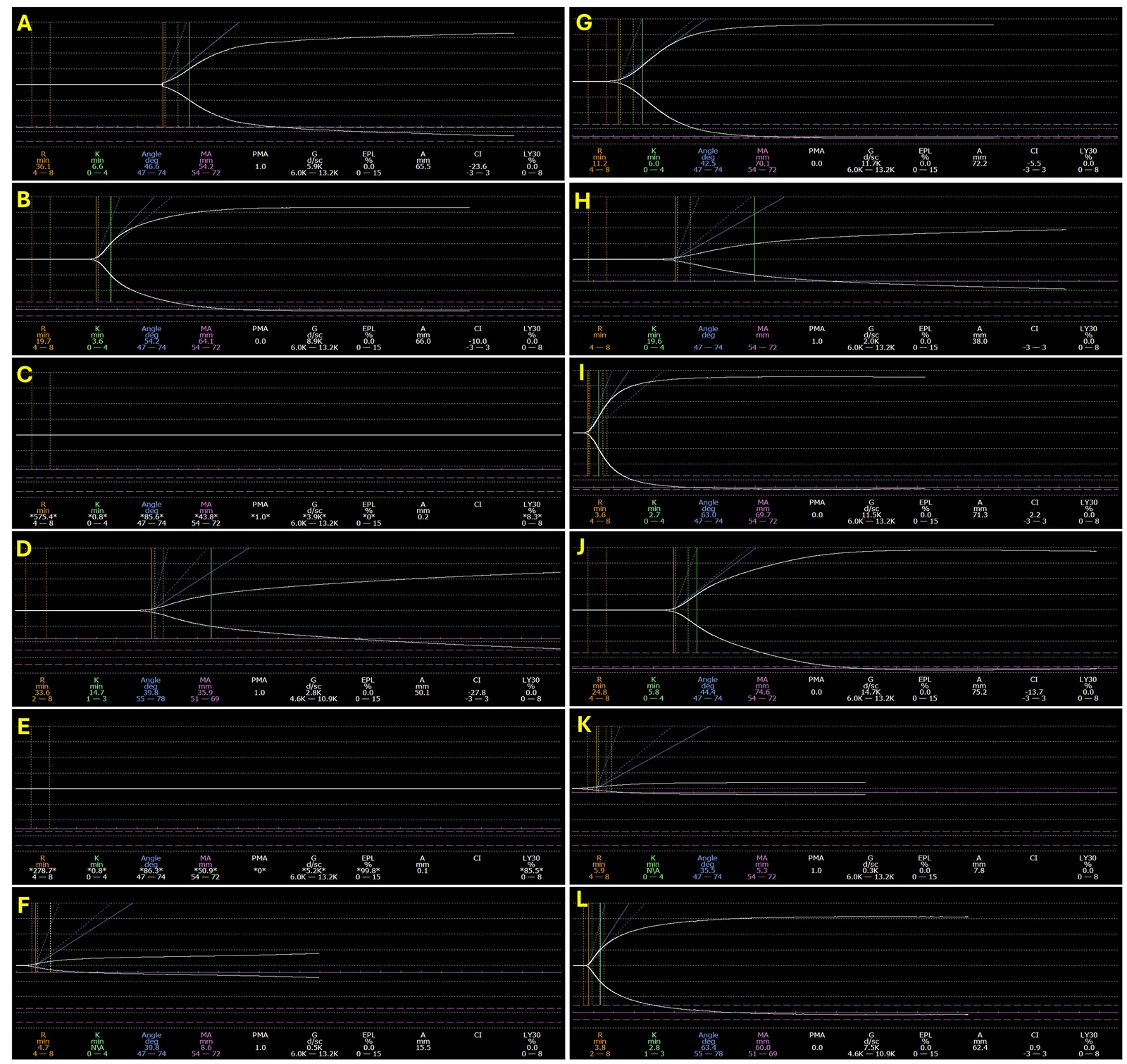
Serial thromboelastography (TEG) evolution during hospitalization. The figure presents a series of thromboelastography (TEG) tracings (panels A-L) illustrating the dynamic evolution of the patient’s hemostatic profile throughout hospitalization. In the early phase (panels A-C), a pattern of severe hypocoagulability is observed, characterized by markedly prolonged reaction time (R), in some instances extremely prolonged, indicating a critical impairment in initial thrombin generation. Additionally, a reduced alpha angle and decreased maximum amplitude (MA) are noted, reflecting both delayed clot formation kinetics and impaired clot strength. This pattern is consistent with a significant defect in the intrinsic coagulation pathway, in line with the presence of a factor VIII inhibitor. In the intermediate phase (panels D-H), the tracings demonstrate a heterogeneous pattern, with persistent abnormalities in clot initiation (prolonged R) and variable clot formation. In some recordings, evidence of hyperfibrinolysis is observed, as reflected by elevated LY30 values, suggesting clot instability and an additional contribution to ongoing clinical bleeding. This phase corresponds clinically to persistent hemorrhage, multiple surgical interventions, and the need for intensive hemostatic therapy. In the late phase (panels I-L), following the initiation of immunosuppressive therapy and targeted hemostatic treatment, a progressive improvement in viscoelastic parameters is observed. There is shortening of the R time, normalization of the alpha angle, and recovery of maximum amplitude (MA), indicating restoration of thrombin generation and clot stability. These changes correlate with the reduction in inhibitor titers and the recovery of factor VIII functional activity. LY30: lysis at 30 minutes

Importantly, despite the administration of bypassing agents such as recombinant activated factor VII (rFVIIa), viscoelastic parameters do not normalize immediately. This reflects the fact that these agents enhance thrombin generation independently of the intrinsic tenase complex but do not correct the underlying immune-mediated mechanism. Therefore, normalization of TEG parameters correlates more closely with inhibitor eradication through immunosuppressive therapy rather than the immediate effect of hemostatic agents, as shown in Table 2.

**Table 2:** Temporal evolution of viscoelastic parameters and their correlation with laboratory findings and therapeutic interventions Thromboelastography (TEG) was used to dynamically assess the progression of the coagulopathy associated with acquired hemophilia Early recordings showed alterations in the initiation phase of coagulation with prolonged reaction time (R), followed by episodes of severe hypocoagulability and hyperfibrinolysis. Integration of viscoelastic parameters with conventional laboratory tests (aPTT, fibrinogen, and D-dimer) and therapeutic interventions allowed continuous monitoring of treatment response, demonstrating progressive restoration of hemostatic function as the factor VIII inhibitor decreased. aPTT: activated partial thromboplastin time; R: reaction time; MA: maximum amplitude; INR: international normalized ratio; FVIII: factor VIII; K: clot formation time; α: alpha angle; TEG: thromboelastography; LY30: lysis at 30 minutes; CI: coagulation index; rFVIIa: recombinant activated factor VII; PCC: prothrombin complex concentrate; FEIBA: factor eight inhibitor bypassing activity

Day	Clinical Evolution	TEG Parameters	Associated Laboratory Findings	Therapeutic Intervention	Pathophysiological Interpretation	Subsequent Evolution
Day 2	Initial coagulation disturbance	R 36.1 min ↑; K 6.6 min ↑; α 46° ↓; MA 54 mm	aPTT 53.8 s; INR 1.08; fibrinogen 535 mg/dL	No intervention	Defect in the initiation phase of coagulation compatible with FVIII inhibitor	Persistent aPTT prolongation
Day 3	Progression of hypocoagulability	R 19.7 min ↑; α 54°; MA 64 mm	aPTT 71.7 s; fibrinogen 573 mg/dL; D-dimer 1085 ng/mL	—	Fibrinolytic activation with still functional coagulation	Progressive aPTT alteration
Day 6	Onset of hemostatic decompensation	R 575 min ↑; α 85°; MA 43 mm ↓; LY30 90%	aPTT >100 s	PCC + fibrinogen + rFVIIa	Severe coagulopathy with marked fibrinolysis	Persistent inability to form a stable clot
Day 6	Critical coagulopathy	R 1193 min; MA 36 mm; severely altered CI	—	Repeated rFVIIa	Near-complete inhibition of thrombin generation	Persistent active bleeding
Day 7	Persistent hypocoagulability	R 33 min; K 14 min; α 39°; MA 35 mm	aPTT 103 s; fibrinogen 443 mg/dL	Rituximab + rFVIIa	Reduced clot propagation	Partial response
Day 8	Initiation of intensive immunosuppression	R 278 min; α 85°; MA 50 mm	Fibrinogen 457 mg/dL	Methylprednisolone + cyclophosphamide + PCC	Partial restoration of clot formation	Improvement in TEG dynamics
Day 10	First TEG showing effective coagulation	R 4 min; α 50°; MA 72 mm; G 13.1	Fibrinogen 391 mg/dL; D-dimer 568	rFVIIa + tranexamic acid	Functional restoration of clot formation	Decrease in bleeding
Day 11	Partial hemostatic stabilization	R 11 min; K 6 min; MA 70 mm	Elevated D-dimer	Continuous antifibrinolytic therapy	Relatively stable clot formation	Favorable clinical evolution
Day 13	New hemostatic deterioration	R 25 min; K 19 min; MA 20 mm	D-dimer 1285 ng/mL	FEIBA + PCC + fibrinogen	Impaired clot propagation	Subsequent recovery
Day 14	Functional recovery	R 3.6 min; α 63°; MA 69 mm	D-dimer 725 ng/mL	Rituximab + rFVIIa	Normalization of coagulation kinetics	Hemostatic control
Day 18	Stabilization phase	R 24.8 min; MA 74 mm	Fibrinogen 338 mg/dL	Intermittent rFVIIa	Functional coagulation with persistent fibrinolytic activation	Progressive improvement
Day 19	Transient clot dysfunction	R 5.9 min; MA 5 mm	Fibrinogen 356 mg/dL	Antifibrinolytic therapy	Transient hypocoagulable episode	Subsequent recovery
Day 26	Final recovery phase	R 3.8 min; K 2.8 min; α 63°; MA 60 mm	Fibrinogen 440 mg/dL; D-dimer 500 ng/mL	Minimal therapy	Stable functional coagulation	Resolution of hemorrhagic event

Diagnosis of acquired hemophilia

Given the persistence of isolated prolongation of aPTT and the clear discordance between the severity of clinical bleeding and the findings expected in coagulopathies associated with obstetric hemorrhage, a targeted diagnostic evaluation was performed to identify possible acquired coagulation inhibitors.

In order to precisely characterize the hemostatic abnormality observed during the patient’s clinical course, serial conventional coagulation studies, patient plasma dilution assays, control plasma (normal pool) dilutions, correction studies (mixing tests), factor VIII activity measurements, and inhibitor quantification using the Nijmegen-modified Bethesda method were performed. The integration of these studies demonstrated that the prolonged aPTT did not correspond to a simple factor deficiency but rather to the presence of an acquired inhibitor directed against factor VIII with concentration-dependent behavior.

During the first days of hospitalization, laboratory testing revealed a marked prolongation of aPTT with relative preservation of prothrombin time (PT), a pattern characteristic of disorders predominantly affecting the intrinsic pathway of coagulation, as shown in Figure 2. To further characterize this abnormality, serial dilutions of the patient’s plasma and control plasma were performed.

**Figure 2:**
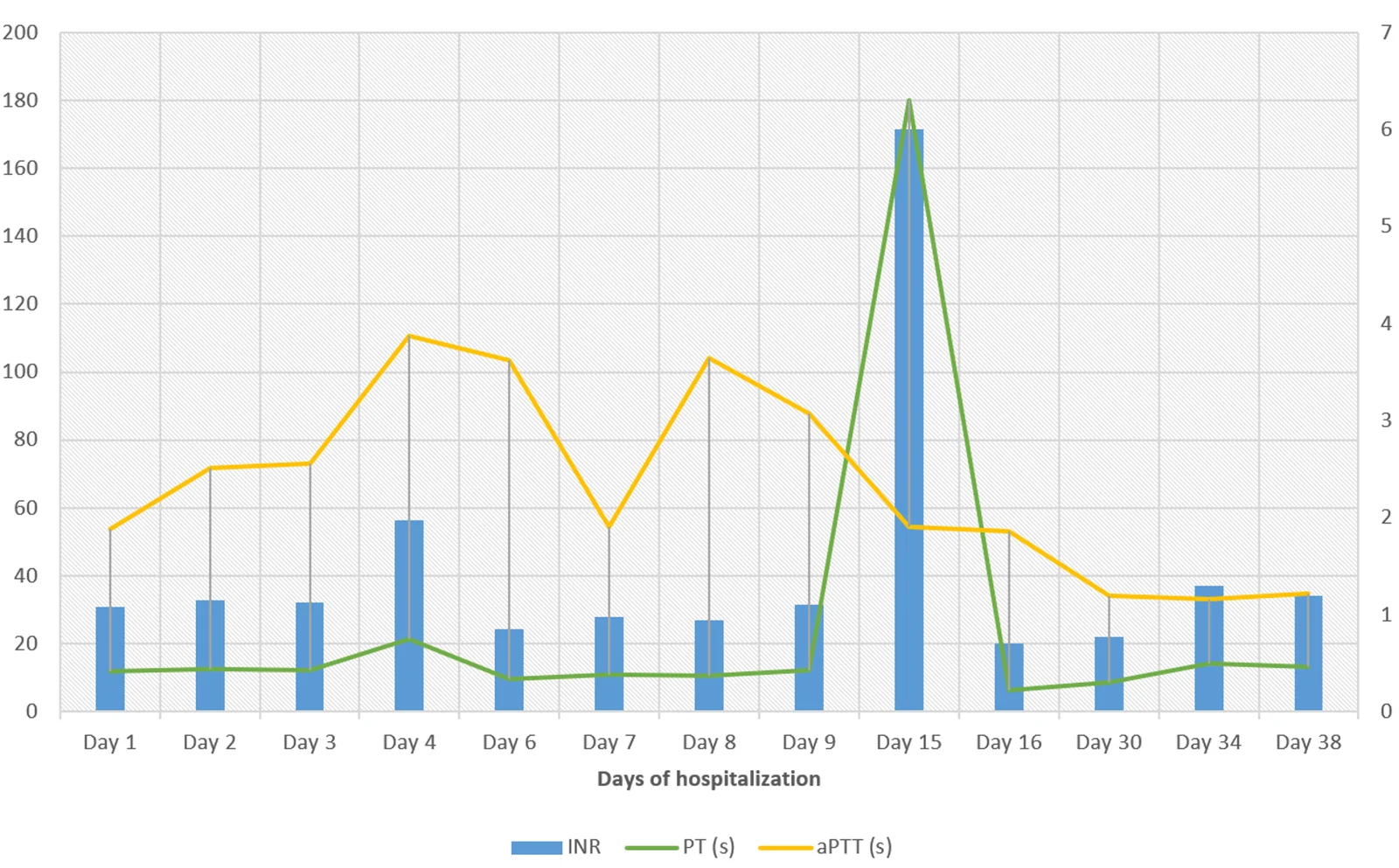
Evolution of coagulation parameters during hospitalization. This figure illustrates the evolution of coagulation parameters during hospitalization, including prothrombin time (PT), international normalized ratio (INR), and activated partial thromboplastin time (aPTT) across different hospital days. During the early phase of hospitalization (Days 1-9), a marked prolongation of aPTT is observed, reaching values of approximately 110 seconds, suggesting a significant disturbance of the intrinsic coagulation pathway. During this period, PT and INR remain relatively stable, with only moderate elevations. The most notable finding occurs around Day 15 of hospitalization, where a sudden increase in PT and INR is observed, reaching extreme values (PT close to 180 seconds and INR greater than 6), indicating an episode of severe coagulopathy. Following therapeutic interventions, a progressive normalization of coagulation parameters can be observed. In the late phase (Days 30-38), PT, INR, and aPTT values show a trend toward stabilization, approaching values closer to the normal range, reflecting a gradual improvement in the patient’s hemostatic status over time. Overall, the temporal evolution demonstrates a severe coagulopathy during the intermediate phase of hospitalization, followed by progressive recovery after hemostatic and immunosuppressive treatment.

Patient plasma dilutions

Dilutions of the patient’s plasma demonstrated a heterogeneous and non-linear behavior, particularly during the early phases of the disease, a finding characteristic of the presence of acquired coagulation inhibitors.

On hospital day 6, the 1:2 dilution showed a PT of 19.4 seconds, an international normalized ratio (INR) of 1.77, and an aPTT of 201.2 seconds, indicating profound alteration of intrinsic coagulation. When dilution was increased to 1:4, PT further prolonged to 31.2 seconds with an INR of 2.93, while the patient’s plasma remained incapable of generating an effective hemostatic response.

On hospital day 9, the 1:2 dilution again showed marked prolongation of aPTT (180 seconds) with PT 18.4 seconds and INR 1.7, whereas at 1:4 dilution PT increased to 33.1 seconds with INR 3.11. This persistent pattern suggested that the hemostatic defect was not corrected simply by plasma dilution, supporting the presence of a circulating inhibitor directed against factor VIII.

On hospital day 14, dilution studies demonstrated a particularly irregular pattern. At 1:2 dilution, PT was >180 seconds with an aPTT of 61.7 seconds, whereas at 1:4 dilution, PT decreased to 9.9 seconds (INR 0.9) but aPTT again prolonged to 111.3 seconds. At higher dilutions (1:8 and 1:16), extremely prolonged aPTT values of up to 260 seconds were documented. This erratic and dilution-dependent behavior is characteristic of acquired inhibitors against coagulation factors, which exhibit complex kinetics and non-linear neutralization, a phenomenon widely described in acquired hemophilia.

By hospital day 20, patient plasma dilutions began to show progressive improvement, in parallel with the immunosuppressive therapy that had been initiated. The 1:2 dilution showed PT 13.6 seconds, INR 1.25, and aPTT 83 seconds, while higher dilutions demonstrated prolongation consistent with the expected mathematical effect of dilution. This pattern suggested partial recovery of hemostatic activity, likely associated with progressive reduction in factor VIII inhibitor titers and recovery of its functional activity.

These findings are illustrated in Figure 3, which shows the behavior curves of coagulation times across different patient plasma dilutions, demonstrating the characteristic variability induced by the presence of an acquired inhibitor. Early dilutions show disproportionate prolongation and non-linear patterns, whereas later stages of treatment demonstrate a trend toward progressive normalization of coagulation parameters.

**Figure 3:**
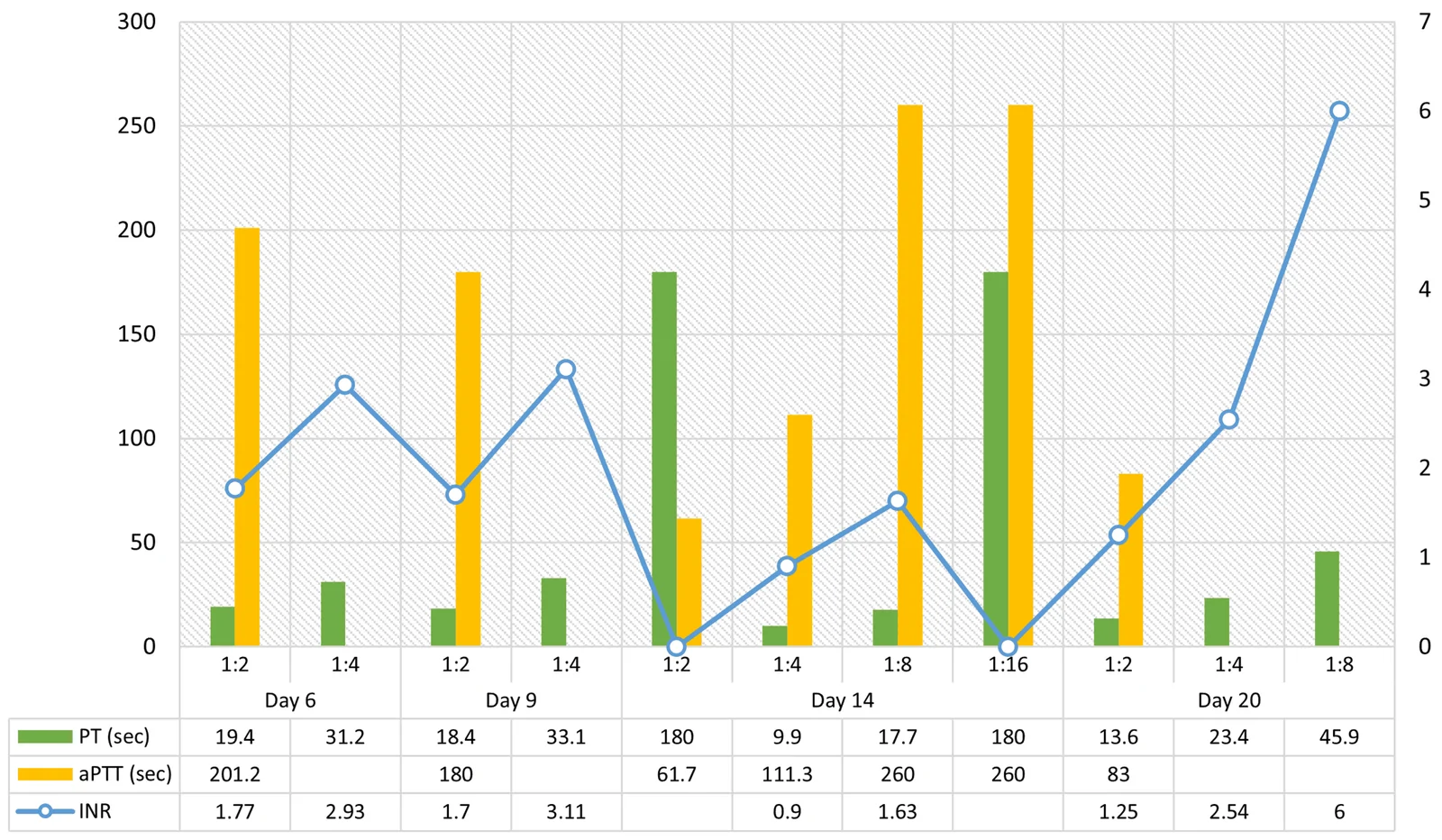
Patient plasma dilution curves showing the evolution of PT and aPTT at different dilution ratios during hospitalization. PT: prothrombin time; aPTT: activated partial thromboplastin time; INR: international normalized ratio

Control plasma dilutions (pool plasma)

Dilutions performed using control plasma (normal pooled plasma) demonstrated the expected behavior of an intact hemostatic system. As the dilution factor increased, both prothrombin time (PT) and activated partial thromboplastin time (aPTT) prolonged progressively and proportionally, reflecting the gradual decrease in the concentration of functional coagulation factors.

This linear and predictable pattern was consistently observed in all determinations performed on hospital days 6, 9, 14, and 20, confirming the analytical stability of the measurement system.

Figure 4 illustrates the evolution of coagulation parameters across different dilutions of control plasma, where a direct relationship between the degree of dilution and prolongation of coagulation times can be observed. This phenomenon is expected when the concentration of plasma coagulation factors decreases in the absence of inhibitors.

**Figure 4:**
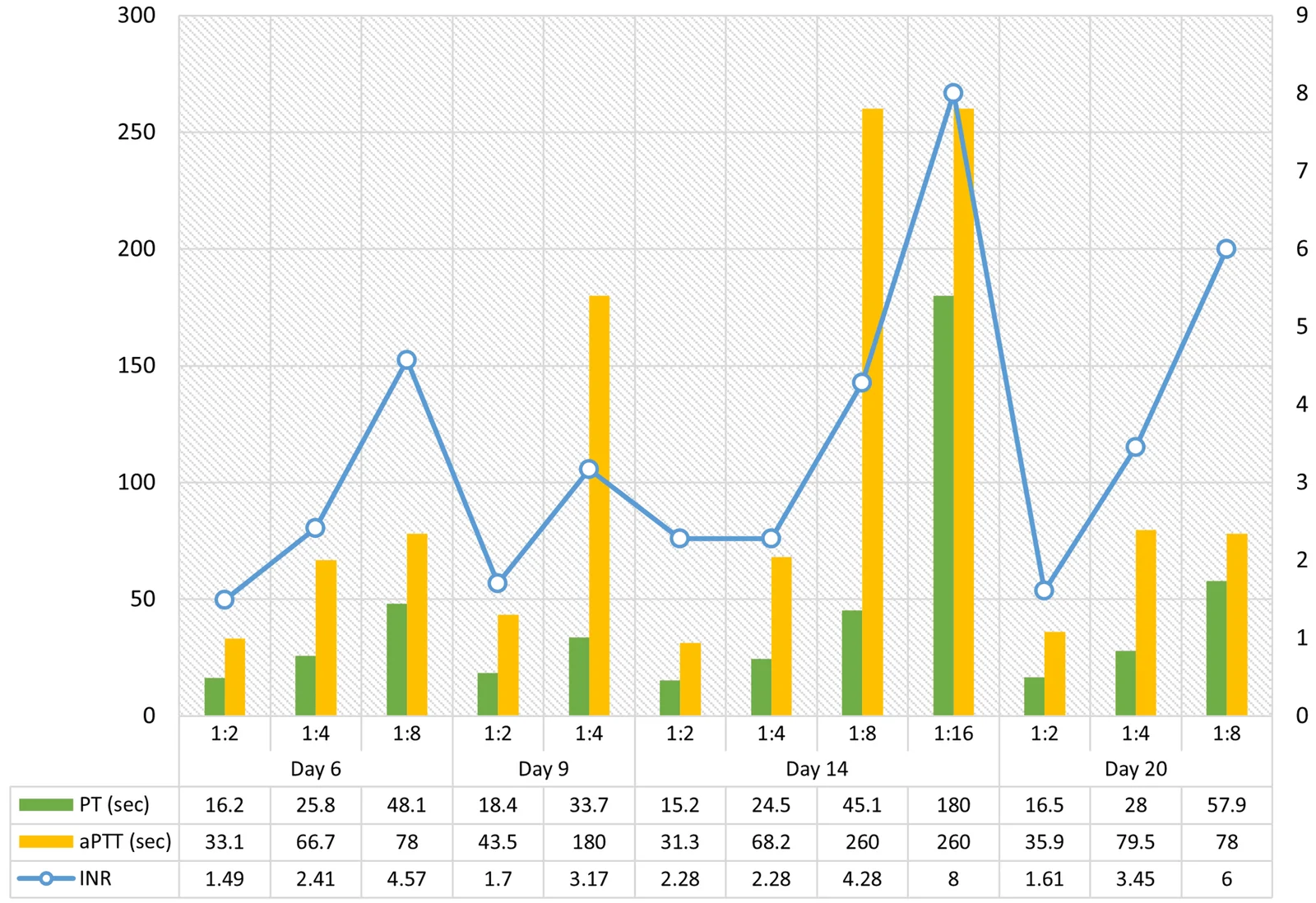
Pool plasma dilution curves showing progressive prolongation of PT and aPTT according to dilution factor, consistent with normal coagulation factor kinetics. PT: prothrombin time; aPTT: activated partial thromboplastin time; INR: international normalized ratio

Comparison between the behavior of the patient’s plasma and control plasma demonstrated that the abnormalities observed in patient plasma dilutions were not attributable to methodological artifacts or analytical variability, but instead reflected an intrinsic property of the patient’s plasma compatible with the presence of a circulating factor VIII inhibitor. While the control plasma showed a linear dilution-dependent prolongation, the patient’s plasma exhibited erratic, non-linear, inhibitor-dependent patterns, a hallmark of the complex kinetics of autoantibodies observed in acquired hemophilia.

Correction studies (mixing test)

Mixing studies performed with normal plasma demonstrated a pattern of partial correction of aPTT, without complete normalization during the early phases of the disease, a finding characteristic of acquired coagulation inhibitors.

On hospital day 6, mixing the patient’s plasma with normal plasma in increasing proportions resulted in a reduction of aPTT from 42.2 seconds at 1:2 dilution to 30.7 seconds at 1:16 dilution. Although progressive shortening of the coagulation time was observed with increasing proportions of normal plasma, complete correction was not achieved at the initial dilutions, suggesting the presence of a circulating inhibitor capable of partially neutralizing factor VIII activity. In simple factor deficiencies, mixing with normal plasma typically results in complete normalization of aPTT from the earliest dilutions.

A similar pattern was observed on hospital day 9, where aPTT decreased from 39.9 seconds at 1:2 dilution to 32.9 seconds at higher dilutions, with residual prolongation persisting. This pattern is consistent with the characteristic kinetics of type II inhibitors, in which an initial neutralization phase is followed by residual factor activity.

By hospital day 20, the mixing test showed a more consistent correction, with aPTT 35.4 seconds at 1:2 dilution and 30.9 seconds at 1:8 dilution, reflecting a progressive reduction in inhibitor activity, likely associated with the effect of the initiated immunosuppressive therapy and the reduction of factor VIII inhibitor titers.

These results are illustrated in Figure 5, which graphically represents the evolution of aPTT across the different mixing test dilutions, demonstrating the transition from initial incomplete correction consistent with a circulating inhibitor toward progressive normalization as the inhibitor burden decreased during treatment.

**Figure 5:**
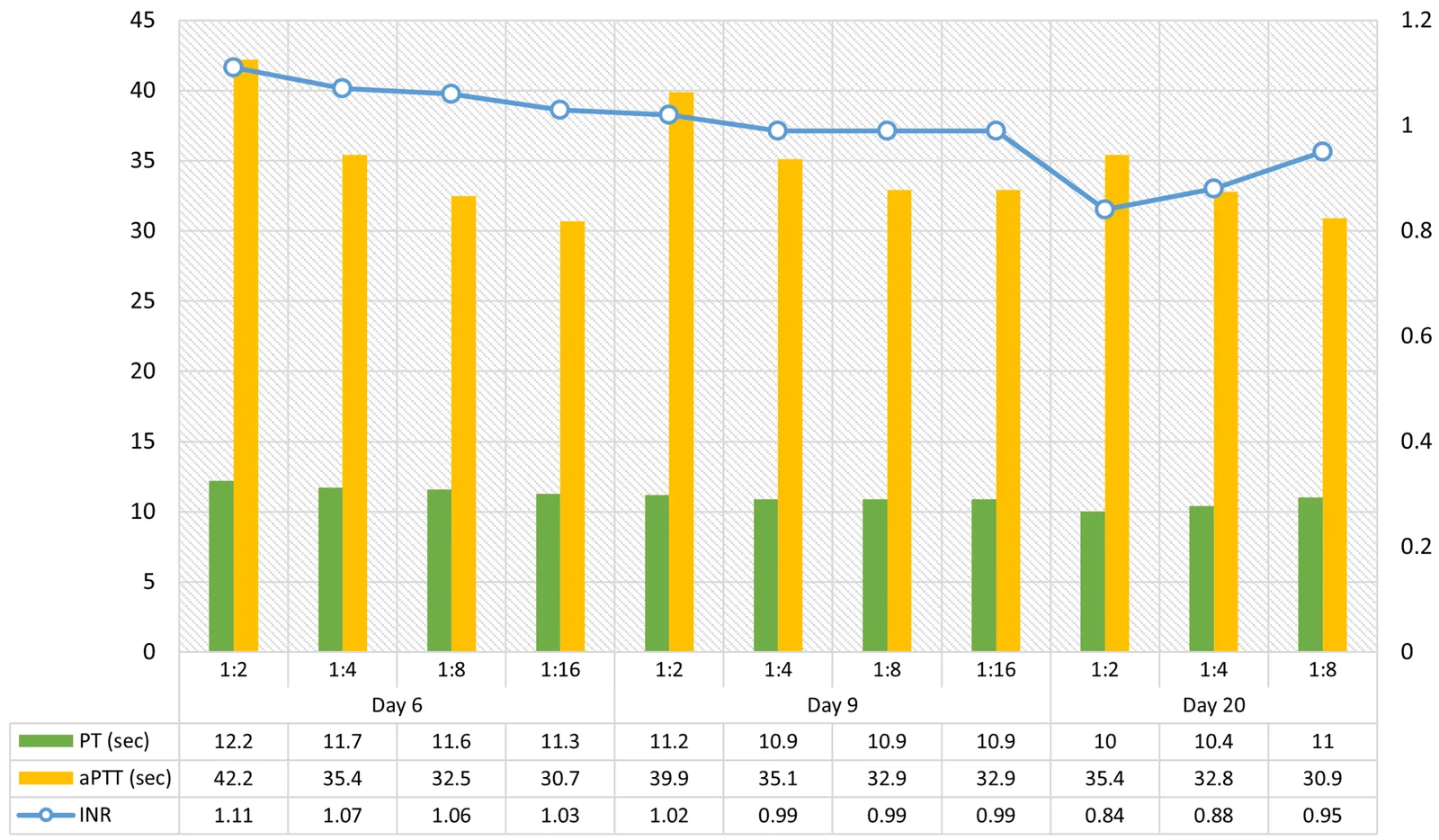
Correction studies (mixing test) showing partial normalization of aPTT with increasing proportions of normal plasma, consistent with the presence of a circulating factor VIII inhibitor. PT: prothrombin time; aPTT: activated partial thromboplastin time; INR: international normalized ratio

Factor VIII activity and inhibitor evolution

Factor VIII (FVIII) activity demonstrated progressive recovery during immunosuppressive therapy, reflecting the gradual eradication of the circulating inhibitor and restoration of hemostatic function. At the onset of the clinical course, on hospital day 6, FVIII activity was markedly decreased (0.78%), far below the physiological range (50-150%), consistent with severe functional inhibition of the intrinsic tenase complex.

During clinical evolution and after initiation of immunosuppressive therapy, a progressive increase in factor activity was observed. On hospital day 11, FVIII activity increased to 7.20%, reflecting partial initial recovery. Subsequently, on hospital day 24, FVIII activity reached 28.20%, indicating more substantial functional recovery of the intrinsic coagulation pathway. Finally, on hospital day 32, FVIII activity reached 55.50%, placing it within the hemostatically effective range and consistent with restoration of coagulation system function.

In parallel, quantification of the factor VIII inhibitor using the Nijmegen-modified Bethesda method showed an inverse evolution. Initially, on hospital day 6, the inhibitor titer was 4.03 BU/mL, confirming the presence of a clinically significant acquired inhibitor. On hospital day 11, a transient increase to 7.4 BU/mL was observed, a phenomenon described during the early phase of immunosuppressive therapy and related to immune system dynamics.

Subsequently, inhibitor titers progressively declined, reaching 0.96 BU/mL on day 16, 0.6 BU/mL on day 24, and finally 0 BU/mL on day 32, indicating complete eradication of the inhibitor.

The inverse relationship between FVIII activity and inhibitor titers during therapy is illustrated in Figure 6, which clearly demonstrates that progressive reduction of the inhibitor was accompanied by gradual recovery of FVIII activity. This dynamic reflects the combined effect of immunosuppressive therapy (corticosteroids, cyclophosphamide, and rituximab) and the hemostatic strategies implemented during the critical bleeding phase.

**Figure 6:**
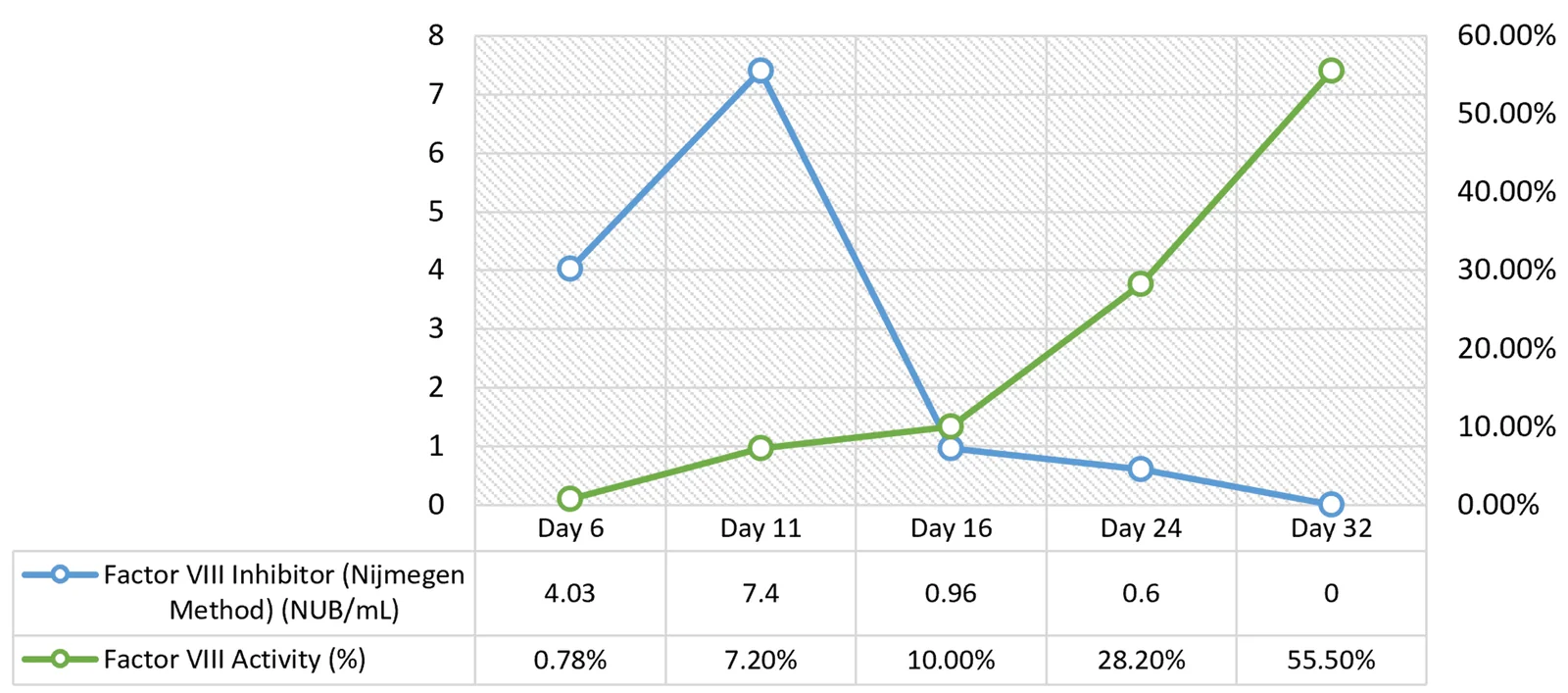
Relationship between factor VIII activity and factor VIII inhibitor during treatment. Inverse evolution between factor VIII activity and inhibitor titer (Nijmegen method) during treatment, demonstrating progressive recovery of hemostatic function as the inhibitor burden decreased.

Overall, these findings demonstrated that the prolongation of aPTT observed in this patient was caused by an acquired inhibitor directed against factor VIII. Dilution and correction studies revealed a concentration-dependent behavior and incomplete correction characteristic of this type of autoantibody. Temporal evolution of the tests further showed that recovery of the hemostatic system coincided with progressive reduction in inhibitor titers and restoration of factor VIII activity following immunosuppressive therapy.

The graphical representations derived from these studies provide a dynamic visualization of the interaction between the inhibitor, factor VIII activity, and coagulation test behavior during treatment, offering additional evidence regarding the pathophysiology and therapeutic response in this case of postpartum acquired hemophilia.

Immunosuppressive treatment and targeted hemostatic therapy

Once the diagnosis of acquired hemophilia secondary to a factor VIII inhibitor was established, a dual therapeutic approach was promptly initiated, aimed at both controlling active bleeding and eradicating the circulating autoantibody. This strategy is essential in acquired hemophilia, as isolated hemostatic management is often insufficient if inhibitor production is not effectively suppressed.

Immunosuppressive therapy was initiated on day 7 of hospitalization and consisted of a combined regimen including high-dose corticosteroids, cyclophosphamide, and rituximab, with the goal of suppressing the autoimmune response responsible for the production of anti-factor VIII antibodies. Corticosteroids provided rapid initial suppression of inflammatory and lymphocytic activation, while cyclophosphamide contributed to inhibiting the proliferation of antibody-producing B lymphocytes. Rituximab, a monoclonal antibody targeting the CD20 antigen on B cells, was administered to induce selective B-cell depletion, thereby reducing the production of neutralizing autoantibodies.

In parallel, targeted hemostatic therapy with bypassing agents was initiated, as recommended in patients with factor VIII inhibitors. These agents enable activation of the coagulation cascade through alternative pathways independent of the intrinsic tenase complex, which was functionally inhibited in this patient. In this context, despite the underlying functional deficiency of factor VIII, recombinant activated factor VII (rFVIIa) was administered - not as a replacement therapy, but as a bypass strategy to restore thrombin generation. rFVIIa promotes direct activation of factor X on the platelet surface, thereby bypassing the need for the factor VIII-factor IX (FVIII-FIX)complex and allowing fibrin formation and clot stabilization even in the presence of circulating inhibitors.

Additionally, activated prothrombin complex concentrate (aPCC) was used as an alternative bypassing agent, providing activated coagulation factors that enhance thrombin generation through non-factor VIII-dependent mechanisms. The sequential and combined use of these agents reflects the need for a stepwise hemostatic strategy in the setting of severe intrinsic pathway inhibition. During the clinical course, administration of bypassing agents was associated with progressive improvement in viscoelastic parameters, particularly a reduction in reaction time (R) on thromboelastography, indicating restoration of thrombin generation.

Adjunctive hemostatic support measures included the administration of tranexamic acid as an antifibrinolytic agent, as well as targeted fibrinogen replacement when clinically indicated. During episodes of severe anemia, packed red blood cell transfusions were administered to maintain adequate oxygen-carrying capacity and hemodynamic stability.

The combination of early immunosuppression, targeted hemostatic therapy with bypassing agents, and supportive transfusion strategies allowed for progressive control of bleeding episodes and stabilization of the patient’s clinical condition, while achieving gradual reduction of inhibitor activity and recovery of factor VIII function.

During the initial phase (days 1-5), a high intensity of treatment was observed, characterized by frequent administration of eptacog alfa (rFVIIa) and tranexamic acid, along with additional hemostatic support including fibrinogen and prothrombin complex concentrate (PCC). During this same period, immunomodulatory therapy was initiated, including rituximab, methylprednisolone, cyclophosphamide, and mesna, reflecting early targeting of the underlying autoimmune mechanism.

In the intermediate phase (days 6-15), continuous antifibrinolytic therapy with tranexamic acid was maintained, while rFVIIa was administered intermittently for control of persistent or recurrent bleeding episodes. During this stage, **FEIBA **(activated prothrombin complex concentrate) was introduced transiently as an additional bypassing agent, indicating the need for escalated hemostatic strategies in the setting of ongoing inhibitor-mediated coagulopathy.

In the late phase (days 16-25), the therapeutic pattern showed a gradual reduction in immunosuppressive interventions, with predominance of targeted hemostatic therapy, mainly rFVIIa and tranexamic acid. However, intermittent peaks of rFVIIa administration were observed, particularly around days 18 and 22, suggesting episodic hemorrhagic exacerbations requiring intensified hemostatic support.

Overall, the temporal pattern reflects a multimodal and stepwise therapeutic approach, in which bypassing agents were used intensively during the early phase and subsequently adjusted according to clinical evolution, while immunosuppressive therapy was concentrated in the initial period to achieve eradication of the underlying inhibitor, as illustrated in Figure 7.

**Figure 7:**
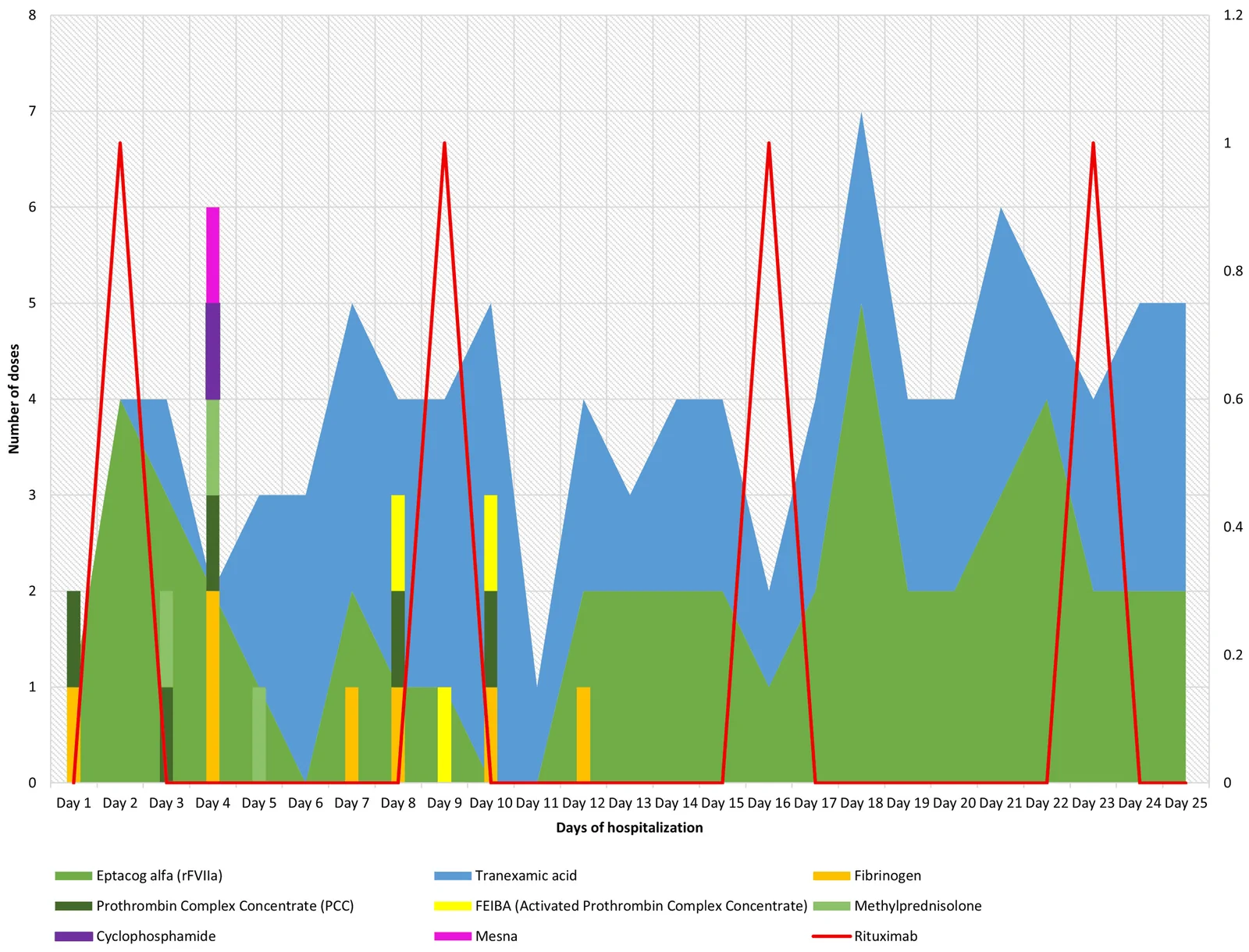
Daily administration of hemostatic and immunosuppressive treatments during hospitalization. This figure illustrates the daily temporal distribution of hemostatic and immunosuppressive therapies administered during the first 25 days of hospitalization, expressed as the number of doses per day. rFVIIa: recombinant activated factor VII; FEIBA:

Hematologic interventions during clinical evolution

The hematologic management of the patient was structured around two fundamental therapeutic pillars: immediate control of hemorrhage through the use of bypassing hemostatic agents and targeted transfusion therapy, and eradication of the factor VIII inhibitor through immunosuppressive treatment. Given that the patient presented with persistent bleeding in the context of acquired hemophilia with critically low factor VIII activity and the presence of a circulating inhibitor, a stepwise and multidisciplinary approach was implemented, with continuous clinical, viscoelastic, and conventional laboratory monitoring.

Initially, bleeding control was based on hemostatic therapies aimed at restoring thrombin generation independently of the intrinsic tenase complex, which was functionally blocked by the inhibitor. For this purpose, bypassing agents were administered, primarily recombinant activated factor VII (rFVIIa) and activated prothrombin complex concentrate (aPCC). These agents were administered alternately during the phases of greatest hemorrhagic activity. Their mechanism of action allowed thrombin generation through the extrinsic pathway, partially compensating for the functional absence of factor VIII.

In parallel, systemic antifibrinolytic therapy was administered, mainly tranexamic acid, with the aim of stabilizing the formed clot and reducing fibrin degradation, particularly in the context of documented hyperfibrinolysis on thromboelastography.

Due to persistent hemorrhage and significant blood loss, the patient required transfusion support with packed red blood cells during the critical phase of bleeding in order to maintain adequate hemoglobin levels and ensure appropriate tissue oxygen delivery and hemodynamic stability.

Simultaneously with hemostatic control, immunosuppressive therapy aimed at eradicating the factor VIII inhibitor was initiated. The treatment regimen included methylprednisolone pulses as initial anti-inflammatory therapy, followed by administration of cyclophosphamide combined with mesna as a cytotoxic immunomodulatory agent. In addition, rituximab, a monoclonal anti-CD20 antibody that induces depletion of B lymphocytes responsible for autoantibody production, was administered. This therapeutic strategy resulted in a progressive reduction in inhibitor titers and gradual recovery of factor VIII activity.

Therapeutic response was monitored through clinical parameters, viscoelastic studies, and specific coagulation assays, demonstrating progressive reduction in bleeding, recovery of factor VIII activity, and complete disappearance of the inhibitor by the end of hospitalization, as shown in Table 3.

**Table 3:** Hematologic interventions administered during hospitalization Bypassing agents (recombinant activated factor VII (rFVIIa) and activated prothrombin complex concentrate (aPCC)) were used for bleeding control, combined with antifibrinolytic therapy and transfusional support. Immunosuppressive therapy with methylprednisolone, cyclophosphamide, and rituximab was initiated for eradication of the factor VIII inhibitor. Hemostatic response was monitored using thromboelastography (TEG), factor VIII activity, and inhibitor titers measured by the Nijmegen Bethesda assay.

Intervention Type	Therapy	Therapeutic Objective	Timing of Use	Clinical Outcome
Bypass agent	Recombinant activated factor VII (rFVIIa)	Direct activation of factor X and thrombin generation independent of factor VIII (FVIII)	Hemorrhagic critical phase	Partial control of bleeding
Bypass agent	Activated prothrombin complex concentrate (aPCC)	Supply of activated coagulation factors (IIa, IXa, Xa) to promote thrombin generation bypassing FVIII	Alternated with rFVIIa during the critical phase	Improvement of hemostasis
Antifibrinolytic therapy	Tranexamic acid	Inhibition of plasminogen activation and stabilization of fibrin clot	During episodes of hyperfibrinolysis	Reduction of clot degradation
Targeted hemostatic therapy	Fibrinogen concentrate	Optimization of fibrin formation guided by viscoelastic testing (thromboelastography (TEG))	During hypocoagulable states	Improvement of clot strength
Transfusion support	Packed red blood cells	Correction of hemorrhagic anemia and maintenance of oxygen delivery	During the hemorrhagic phase	Progressive hemoglobin recovery
Immunosuppressive therapy	Methylprednisolone pulses	Initial suppression of autoimmune response	At diagnosis	Reduction of inflammatory immune activity
Immunosuppressive therapy	Cyclophosphamide with mesna	Inhibition of proliferation of autoreactive B lymphocytes producing FVIII inhibitors	Early treatment phase	Progressive decrease of inhibitor levels
Biologic therapy	Rituximab (4 doses)	Depletion of CD20-positive B cells producing FVIII autoantibodies	During hospitalization	Complete inhibitor eradication

Clinical course and hematologic recovery

Following the initiation of immunosuppressive treatment and targeted hemostatic therapy, the patient showed progressive improvement in both clinical and laboratory parameters, with gradual reduction in hemorrhagic episodes and stabilization of her hemodynamic status.

From a hematologic standpoint, a progressive increase in hemoglobin levels was observed after control of active bleeding and transfusional support, allowing gradual recovery of circulatory stability. In parallel, serial coagulation studies demonstrated progressive normalization of aPTT, in accordance with the decline in factor VIII inhibitor activity.

Follow-up of inhibitor titers using the Nijmegen method demonstrated a sustained decrease in inhibitory activity, reaching complete negativization around hospital day 32. This phenomenon was accompanied by progressive recovery of factor VIII activity, which ultimately reached levels compatible with effective hemostatic function. Simultaneously, viscoelastic studies using TEG showed progressive improvement in coagulation parameters, reflected by gradual normalization of reaction time, improvement in clot formation kinetics, and recovery of maximum amplitude, indicating restoration of clot stability. In parallel with hemostatic control, the infectious complications of the abdominal wall were managed with serial surgical debridement procedures and local wound care, achieving progressive control of the infectious process and gradual wound healing.

Finally, after several weeks of intensive multidisciplinary treatment, the patient evolved toward complete hematologic remission, characterized by the absence of new hemorrhagic events, negativization of the factor VIII inhibitor, and functional recovery of the hemostatic system. This clinical outcome allowed definitive stabilization of the patient and her subsequent transition to outpatient management under specialized follow-up.

## Discussion

Acquired hemophilia (AH) is a rare bleeding disorder characterized by the development of neutralizing autoantibodies directed against factor VIII (FVIII), leading to severe coagulation impairment and a high risk of potentially life-threatening hemorrhage. Unlike congenital hemophilia, this entity occurs in previously healthy individuals and is often associated with autoimmune diseases, malignancies, medications, or the peripartum period [5,10].

The estimated incidence of acquired hemophilia is approximately 1 to 1.5 cases per million inhabitants per year, with a bimodal distribution that mainly affects older adults and women in the early postpartum period [10]. In the latter group, the disease accounts for approximately 7-21% of reported cases, generally manifesting between 1 and 4 months after delivery, although cases occurring in the immediate puerperium have also been reported [14]. The present case illustrates a particularly complex presentation in which persistent bleeding following an obstetric event was initially attributed to surgical and obstetric causes, delaying recognition of the underlying hemostatic disorder.

From a pathophysiological perspective, acquired hemophilia results from the production of polyclonal IgG4 autoantibodies directed against functional epitopes of factor VIII, particularly in the A2 and C2 domains of the molecule. These antibodies neutralize the cofactor function of FVIII within the intrinsic tenase complex, interfering with factor X activation and significantly reducing thrombin generation [7]. Unlike the antibodies seen in congenital hemophilia with inhibitors, the autoantibodies in acquired hemophilia usually exhibit type II kinetics, characterized by partial but persistent inhibition of factor VIII activity, which explains the presence of residual FVIII levels even in patients with high inhibitor titers [10].

In the postpartum setting, the appearance of autoantibodies against factor VIII has been linked to immune dysregulation associated with the immunologic changes of pregnancy and the puerperium. During gestation, expansion of regulatory T lymphocytes (Tregs) promotes maternal-fetal immune tolerance. After delivery, a rapid immune reconstitution may favor activation of autoreactive B-cell clones. This process involves immunologic mediators such as BAFF (B-cell activating factor), anti-inflammatory cytokines such as IL-10, and expansion of autoreactive memory B cells, all of which may contribute to sustained autoantibody production against self-antigens, including factor VIII [14,15]. These immune mechanisms help explain the well-documented association between acquired hemophilia and states of immune activation such as pregnancy, autoimmune diseases, or malignancy.

Clinically, acquired hemophilia is characterized by extensive spontaneous bleeding, unlike congenital hemophilia, in which joint hemorrhages predominate. The most frequent bleeding sites include soft tissues, muscles, skin, the gastrointestinal tract, and the genitourinary tract, whereas hemarthrosis is relatively uncommon. In the obstetric setting, patients may present with persistent bleeding from surgical wounds, prolonged uterine bleeding, or extensive hematomas, as occurred in this case.

The diagnosis of acquired hemophilia requires a high index of clinical suspicion. The most frequent initial laboratory finding is isolated prolongation of aPTT with normal PT and platelet count. This pattern reflects specific interference with the intrinsic coagulation pathway [5]. In the present case, the persistent prolongation of aPTT was initially interpreted within the context of coagulopathy associated with massive obstetric hemorrhage, a situation that may delay diagnosis in critically ill patients.

Mixing studies represent a key diagnostic step for differentiating between coagulation factor deficiencies and circulating inhibitors. In the presence of factor deficiency, mixing patient plasma with normal plasma usually normalizes the aPTT. In contrast, when a circulating inhibitor is present, correction is typically incomplete or transient, a phenomenon observed in this case [15].

Definitive diagnosis is established through measurement of factor VIII activity and quantification of the inhibitor using the Bethesda assay or its Nijmegen modification, which is currently considered the reference standard for measuring inhibitors against coagulation factors [5]. In this patient, an initial FVIII activity of less than 1% together with a measurable inhibitor confirmed the diagnosis of acquired hemophilia.

In critically ill patients with complex bleeding, viscoelastic tests such as TEG may provide additional information regarding the global dynamics of coagulation. Although these tests do not specifically identify factor VIII inhibitors, they may show characteristic patterns of altered thrombin generation and clot stability. In this case, serial TEG studies revealed extreme prolongation of reaction time (R), consistent with a significant defect in initial thrombin generation. Episodes of hyperfibrinolysis were subsequently observed, likely reflecting instability of the hemostatic system in the setting of massive bleeding and transfusion therapy [16].

Treatment of acquired hemophilia has two primary goals: control active bleeding and eradicate the inhibitor. For hemostatic control, bypassing agents such as recombinant activated factor VII (rFVIIa) and activated prothrombin complex concentrate (aPCC) are used, as they enable thrombin generation through alternative pathways independent of factor VIII. Several studies have demonstrated bleeding control rates exceeding 80-90% when these agents are administered early [5].

With regard to inhibitor eradication, immunosuppressive therapy is the therapeutic cornerstone. The most commonly used strategies include corticosteroids, cyclophosphamide, and rituximab, administered alone or in combination depending on clinical severity and inhibitor titer. Rituximab has gained increasing importance due to its ability to induce depletion of B lymphocytes that produce autoantibodies, achieving high remission rates in patients with refractory acquired hemophilia [8,17].

The prognosis of acquired hemophilia has improved significantly in recent decades thanks to earlier recognition and the use of modern hemostatic and immunosuppressive therapies. Nevertheless, mortality may still reach 8-22%, mainly due to severe hemorrhage or infectious complications associated with immunosuppressive treatment [18].

This case illustrates several relevant aspects of this entity. First, it highlights the diagnostic difficulty of acquired hemophilia in patients with obstetric hemorrhage and multiple surgical interventions, in whom persistent bleeding may initially be attributed to mechanical causes. Second, it demonstrates the usefulness of a systematic diagnostic approach that includes mixing studies, coagulation factor assays, and viscoelastic testing. Finally, it underscores the importance of multidisciplinary management involving intensivists, hematologists, and surgeons, which allowed bleeding control, inhibitor eradication, and complete hemostatic recovery.

In summary, postpartum acquired hemophilia represents an uncommon but potentially devastating cause of persistent bleeding during the peripartum period. Early recognition of isolated aPTT prolongation, timely performance of mixing studies, and confirmation through inhibitor measurement are essential for establishing the diagnosis. Prompt treatment with bypassing agents and targeted immunosuppression allows bleeding control and inhibitor eradication in most cases.

## Conclusions

Acquired hemophilia is an uncommon but potentially devastating condition that should be considered in the differential diagnosis of persistent bleeding in previously healthy patients, particularly in the context of the puerperium and complicated obstetric events. The presence of isolated prolongation of activated partial thromboplastin time (aPTT) in a clinical setting of disproportionate bleeding should prompt early suspicion of an acquired inhibitor-mediated coagulation disorder. The present case illustrates how acquired hemophilia may initially manifest as persistent postoperative hemorrhage following cesarean section, which may delay diagnosis when bleeding is attributed to obstetric or surgical causes. In this context, the timely performance of mixing studies, measurement of factor VIII activity, and inhibitor quantification using the Nijmegen method is essential to confirm the diagnosis and guide appropriate treatment. This case also highlights the complementary role of viscoelastic tests such as thromboelastography, which can provide dynamic information on clot generation and stability in critically ill patients with complex bleeding, allowing a better understanding of the underlying hemostatic pathophysiology.

Successful management of this entity requires a multidisciplinary approach, integrating critical care, surgery, hematology, and transfusion support. The combination of bypassing agents for bleeding control and targeted immunosuppressive therapy for inhibitor eradication constitutes the basis of treatment, enabling hemostatic control and hematologic remission in most cases. Finally, this case underscores the importance of maintaining a high index of clinical suspicion in patients with persistent bleeding and prolonged aPTT, since early recognition of acquired hemophilia may significantly alter prognosis and prevent repeated surgical interventions or potentially fatal complications. Timely identification and treatment of this condition allow progressive recovery of factor VIII activity, negativization of the inhibitor, and complete resolution of the hemorrhagic syndrome.
